# Supplementary material for: Adiponectin Intervention to Regulate Betatrophin Expression, Attenuate Insulin Resistance and Enhance Glucose Metabolism in Mice and Its Response to Exercise
Source: Int J Mol Sci. 2022 Sep 13;23(18):10630. doi: 10.3390/ijms231810630 (PMC9505482; doi:10.3390/ijms231810630)
Supplement: Supplementary file 1 [file ijms-23-10630-s001.zip › ijms-1881965-supplementary.pdf]

Table S1. **PCR Primer sequence**

| Gene | FORWARD PRIMER | REVERSE PRIMER |
|---|---|---|
| Lkb1 | CAGTATGACTGTAGTGCCCTAC | CGTCCTCAATGTCAAACAAGTC |
| Adipoq | CCAATGTACCCATTCGCTTTAC | GAAGTAGTAGAGTCCCGGAATG |
| betatrophin | AATCTGCCTGGATGGAACTG | CTGCGTCTGTCTCTGCTCTG |
| PGC1-α | GGATATACTTTACGCAGGTCGA | CGTCTGAGTTGGTATCTAGGTC |
| Tubulin | CAGCGATGAGCACGGCATAGAC | CCAGGTTCCAAGTCCACCAGAATG |
